# A pilot point-of-care kidney disease clinic in primary care to pharmacologically optimise people with chronic kidney disease (PROTECT KIDNEY)

**DOI:** 10.1093/fampra/cmaf083

**Published:** 2025-10-30

**Authors:** Rouvick Mariano Gama, Kathryn Griffiths, Nathan Beencke, Kathryn Dalrymple, Stephanie Mitchell, Prema Ravi, Joseph Mayhew, Sharlene Greenwood, Kate Bramham

**Affiliations:** Department of Inflammation Biology, Faculty of Life Sciences and Medicine, King's College London, London SE5 9NU, United Kingdom; King's Kidney Care, King's College Hospital, King's College Hospital NHS Foundation Trust, London SE5 9RS, United Kingdom; King's Kidney Care, King's College Hospital, King's College Hospital NHS Foundation Trust, London SE5 9RS, United Kingdom; Department of Women and Children’ Health, Faculty of Life Sciences and Medicine, King's College London, London SE5 9RJ, United Kingdom; Department of Long-Term Conditions, Health Innovation Network South London, London SE1 7EU, United Kingdom; Department of Nutritional Sciences, Faculty of Life Sciences and Medicine, King's College London, London SE1 9NH, United Kingdom; King's Kidney Care, King's College Hospital, King's College Hospital NHS Foundation Trust, London SE5 9RS, United Kingdom; Chelsfield Surgery, Orpington Primary Care Network, Orpington BR6 6HD, United Kingdom; Department of Long-Term Conditions, Clinical Effectiveness South East London (CESEL), South East London ICS, 160 Tooley Street, London SE1 2TZ, London; King's Kidney Care, King's College Hospital, King's College Hospital NHS Foundation Trust, London SE5 9RS, United Kingdom; King's Kidney Rehab Team, King's College Hospital, King's College Hospital NHS Foundation Trust, London SE5 9RS, United Kingdom; King's Kidney Care, King's College Hospital, King's College Hospital NHS Foundation Trust, London SE5 9RS, United Kingdom; Department of Women and Children’ Health and Centre for Nephrology, Urology and Transplantation, Faculty of Life Sciences and Medicine, King's College London, London SE5 9RJ, United Kingdom

**Keywords:** chronic kidney disease, sodium-glucose-cotransporter-2 inhibitors, point-of-care, primary care

## Abstract

**Background:**

Chronic kidney disease (CKD) is increasing in prevalence and is associated with substantial morbidity and mortality. Early initiation of cardiorenal protective medications is recommended to improve outcomes. Barriers to implementation include renal function monitoring and resources to initiate and titrate treatment. We aimed to evaluate the feasibility and acceptability of a protocolled point-of-care testing (POCT) pathway to optimise people living with proteinuric CKD in primary care.

**Method:**

A pilot quality improvement study conducted across three general practices in Greater London, United Kingdom. Inclusion criteria were adults (18–80 years) with hypertension and/or type 2 diabetes mellitus, proteinuria, and reduced kidney function (eGFR 30–75 ml/min/1.73m^2^), who were identified using electronic health records. POCT for creatinine and potassium enabled real-time decision-making using a traffic light clinical decision support system. The primary outcome was recruitment rate and patient acceptability. Secondary efficacy outcomes included medication optimisation and renal function changes.

**Results:**

Twenty-five (52%) of 48 patients agreed to participate. Overall, 23/25 (92%) completed the pathway and 20/25 (80%) achieved pharmacological optimisation. There were no significant adverse events. POCT was successful in 44/57 (77%) of cases and well tolerated by most participants (10/13; 77%). Patient satisfaction was high (12/13; 92%), with most preferring advanced nurse practitioners or pharmacists in future clinics.

**Conclusion:**

A POCT-led CKD optimisation pathway is feasible and well-accepted in primary care. While high medication optimisation rates were achieved, barriers to recruitment and engagement remain. Future studies should evaluate scalability, long-term clinical impact, and cost-effectiveness to inform wider implementation.

Key messages

- A traffic-light protocol supported safe titration of medications by health professionals.
- Point-of-care testing was well tolerated and supported patient understanding and engagement.
- The point-of-care kidney clinic is a feasible option to optimise cardio-renal medications.
- This pilot highlights a potential framework to improve access and efficiency in kidney disease.
- Further upscaled evaluation is required to determine cost-effectiveness and sustainability.

## Introduction

Chronic kidney disease (CKD) is increasing in incidence, affecting approximately 7–13% of the population worldwide, and is disproportionately overrepresented in underserved populations [1]. With a prevalence of 36.9% for those over 60 years old, 40% of people with CKD stage 3 or greater living with two or more chronic conditions and direct costs to the National Health Service (NHS) of £6.4 billion per year, CKD poses a growing public health challenge in the UK that requires urgent attention [2].

The risks associated with CKD are amplified in the presence of proteinuria, and therefore, early identification and classification are vital to initiate timely management. Over the last decade, there has been considerable progress in treatments for proteinuric CKD, with strong evidence supporting the role of treatments such as sodium-glucose-costransporter-2 inhibitors (SGLT2i), alongside the established management of angiotensin-converting enzyme inhibitors (ACEi) and/or angiotensin-receptor blockers (ARB), which are both collectively grouped as renin-angiotensin-aldosterone system inhibitors (RAASi) [3–7]. The emergence of SGLT2i has the potential to revolutionise treatment with studies demonstrating in diabetic and non-diabetic patients 29% decreased risk of cardiovascular events, 13%–31% decreased risk of all-cause mortality and a reduction decline in eGFR 1.37–1.92 ml/min/1.73 m^2^ per year [3, 4]. In addition, commencing these treatments for patients with early CKD Stage 3A and diabetes mellitus (i.e. eGFR 60 ml/min/1.73m^2^) could delay needing dialysis by up to 20 years [8].

Consequently, the National Institute for Health and Care Excellence (NICE) CKD guidelines recommend SGLT2i to be prescribed with a maximally tolerated dose of ACEi or ARB [9–12]. However, these medications require renal function monitoring after initiation and dose changes, which is a barrier to implementation, particularly in overstretched and under-resourced health services. Currently, in the UK primary care, the pharmacological management of CKD typically relies on standard laboratory-based blood testing, with patients attending phlebotomy appointments separately from clinical consultations. Identification of patients with CKD is dependent on coding practices and varies substantially. Medication decisions, such as initiating or titrating reno-protective therapies, are often delayed while waiting for laboratory results. They may also be missed, for example, due to different healthcare professionals reviewing results. Overall, this leads to multiple visits and creates additional barriers to timely optimisation [13].

Point-of-care testing (POCT) offers a potential solution. The National Institute for Health and Research (NIHR) Horizon Report in 2014 highlighted POCT as a potential for enhancing current standards of care in CKD [14]. POCT has been described in previous studies, enabling improved detection of CKD, monitoring of kidney function in high-risk groups and those on renally excreted medications and instant screening prior to interventions [15–22]. However, POCT has not previously been used to target rapid optimisation of CKD.

Therefore, we aimed to co-develop and evaluate the feasibility of delivering a protocolled POCT pathway for RAASi and SGLT2i optimisation in primary care, assessed through recruitment and test success rates and to assess acceptability to patients, assessed via validated questionnaire responses and free-text feedback.

## Methods

The development of this pathway and its outcomes was guided by the Medical Research Council Framework for developing and evaluating complex interventions, which emphasises iterative development, feasibility testing, implementation and evaluation [23]. The pathway planning was co-developed with an iterative process involving a stakeholder group comprised of 14 individuals, which included two general practitioners (GPs), two with implementation science expertise, one academic nephrologist with expertise in digital health and tackling health inequalities in kidney disease, two specialist trainee nephrologists, one social worker with expertise in health prevention, one consultant nurse and five patients with lived experiences (who formed the patient and public involvement group). We describe a pilot quality improvement and implementation project created in collaboration with Southeast London Integrated Care Services, Clinical Effectiveness South London (CESEL), King's College Hospital and King's College London.

### Project design and target population

The pilot was run across three primary care practices in Greater London. The target population was people with hypertension and/or type 2 diabetes mellitus, proteinuria and reduced kidney function. There was no formal target sample size set in advance, as recruitment was dependent on the size of the eligible patient population identified during screening in participating practices.

The primary care practices varied in size and setting and had no prior experience running dedicated CKD optimisation clinics. Baseline rates of CKD prevalence and coding varied between practices and relied on standard laboratory-based renal monitoring prior to the pilot pathway. The EPOC Blood Analysis System POCT devices were provided specifically for this project and were not part of routine practice.

The work plan for staff was redistributed to allow participation in the pilot. Staff availability influenced clinic scheduling, in particular meaning clinics had to run in standard working hours. Patients who participated in the pilot were booked in by healthcare professionals involved in the pathway. Administrative staff were informed of the clinic to signpost patients to the clinic rooms, but they had no direct involvement in the administration of the clinic process.

### Inclusion and exclusion criteria

Inclusion criteria were adults (18 years or older) with an estimated glomerular filtration rate (eGFR) between 30 and 75 ml/min/1.73 m^2^, with a urinary albumin: creatinine ratio ≥ 3 mg/mmol if diabetic or ≥ 22.6 mg/mmol if non-diabetic. In addition, they would not already be pharmacologically optimised, which is defined as patients not receiving the maximally tolerated dose of ACEi or ARB and an SGLT2i without any documented allergies or contraindications. Inclusion criteria were directed by NICE recommendations for prescribing SLGT2i (dapagliflozin and empagliflozin) [10, 11].

Exclusion criteria were relative contra-indications to SGLT2i (e.g. Type 1 Diabetes Mellitus, history of Diabetic Ketoacidosis), renal conditions with limited evidence and/or unlicenced indications for SGLT2i (Autosomal dominant polycystic kidney disease, renal transplant or other renal replacement therapy), patient under nephrology services, patients with an eGFR < 30 ml/min/1.73 m^2^ (due to increased potential risk of hyperkalaemia), most recent serum potassium > 5.0 mmol/L, contra-indication to finger prick testing and previous allergy or intolerance to SGLT2i and/or RAASi.

### Identification and screening of target population

In the pilot pathway, target screening was performed using the Active Patient Link (APL) CKD tool, developed by Queen Mary's University London [24]. Shortlisted patients had electronic health records screening to assess for eligibility. Eligible patients were designated with a treatment intent (SGLT2i only; RAASi only; RAASi up-titration and SGLT2i initiation; RAASi and SGLT2i initiation and up-titration). Eligible patients were contacted by a member of the intervention team and invited to participate.

### Point-of-care testing: device and procedure

During each visit, patients had a POCT; either by a finger-prick capillary sample taken using a 23-gauge lancet and collected using a care-fill capillary tube or standard venepuncture. The sample was analysed by EPOC Blood Analysis System (Siemens Healthineers, Erlangen, Germany) to obtain a potassium concentration, creatinine concentration and eGFR using the Chronic Kidney Disease Epidemiology Collaboration (CKD-EPI) 2009 equation without ethnicity co-efficient (calculated with creatinine, age and biological sex).

### Protocol development and validation

The protocol was divided into three domains, based on KDIGO and NICE recommendations: blood pressure, change in creatinine and potassium concentration (Supplementary Figure S1).

Capillary and venous samples for POCT-creatinine (POCT-Cr) and POCT-potassium (POCT-K) using the EPOC Blood Analysis System were validated against the local reference laboratory to determine the accuracy and precision of thresholds for the protocol. Participants were included from a preliminary service evaluation, conducted at a single tertiary renal unit in London, United Kingdom.

Out of 117 patients who participated, 65 (55.6%) had capillary blood samples and 52 (44.4%) venous. The majority were male (*n* = 70, 59.8%), and the mean age was 56.9 ± 15.0 years. The ethnic groups were diverse, with 47 (40.2%) White, 43 (36.8%) Black, 20 (17.1%) South Asian and the remaining 7 (6.0%) from Mixed or Other backgrounds. Median laboratory creatinine was 158 (IQR 113–299) micromol/L, eGFR 35 (IQR 17–57) ml/min/1.73m^2^ and mean potassium was 4.6 ± 0.6 micromol/L. Ninety-three (79.5%) had CKD stage 3 or greater.

There was a very strong positive association between POCT-Cr tests and the laboratory test (Pearson Correlation coefficient: Capillary = 0.935; Venous = 0.995; *P*-values < 0.001). Compared to the reference laboratory test, median bias for capillary and venous POCT-Cr was −11.0 (−19.0 to −4.0) micromol/L and −1.0 (−7.3 to 3.3) micromol/L, respectively.

**Figure 1.**
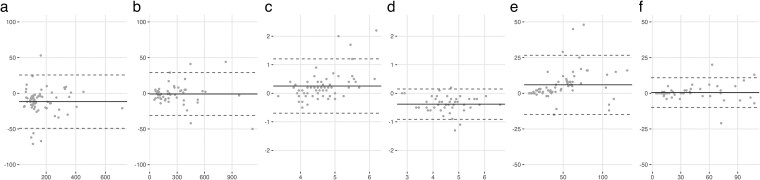
Bland Altman plots illustrating agreement between laboratory and EPOC Blood Analysis System analytes. *X*-axis measures analyte concentration (creatinine, potassium and eGFR) and Y-axis shows the measured difference (solid line), with 95% limits of agreement (dashed line). Illustrations show agreement for serum creatinine concentrations, calculated using IDMS-traceable enzymatic assay and (a) Capillary POCT creatinine and (b) Venous POCT creatinine; serum potassium concentrations and (c) Capillary POCT potassium and (d) Venous POCT potassium; eGFR (CKD-EPI 2009 equation) calculated using age, sex and creatinine from the laboratory results compared to (e) POCT capillary and (f) POCT venous samples.

POC-K were also strongly positively correlated with laboratory testing (Spearman correlation coefficient: Capillary = 0.765; Venous = 0.936). Mean bias for capillary and venous POC-K was 0.25 ± 0.49 mmol/L and −0.4 ± 0.3 compared to laboratory testing. Bias is summarised in Table 1. Agreement is illustrated in the Bland Altman plots in Fig. 1.

**Table 1.** Table showing agreement between laboratory tests for creatinine, potassium and eGFR and POCT using the EPOC blood analysis system for capillary and venous samples.

	Bias	Percentage bias, %	Correlation coefficient	95% Limits of Agreement	*p*-value
Median Capillary Cr (IQR), micromol/L	−11 (−19 to −4)	−7.6 (−11.7 to −2.2)	0.935	−49.1 to 25.6	<0.001
Median Venous Cr (IQR), micromol/L	−1 (−7.3 to 3.3)	−0.4 (−3.6 to 2.1)	0.995	−31.0 to 28.9	<0.001
Mean Capillary Potassium ± SD, mmol/L	0.25 ± 0.49^a^	5.6 ± 10.7	0.765^b^	−0.71 to 1.21	<0.001
Mean Venous Potassium ± SD, mmol/L	−0.38 ± 0.27^a^	− 8.0 ± 5.5	0.936^b^	−0.91 to 0.15	<0.001
Median Capillary eGFR (IQR), mlL/min/1.73m^2^	3 (1 to 8)	10 (3 to 16)	0.954	−14.8 to 26.7	<0.001
Median Venous eGFR (IQR), mlL/min/1.73m^2^	0 (−1 to 2)	0 (−3 to 6)	0.991	−9.9 to 10.9	<0.001

Abbreviations: POCT, Point-of-care testing; Cr, creatinine; eGFR, estimated glomerular filtration rate; IQR, interquartile range; SD, standard deviation.

^a^Mean bias ± standard deviation.

^b^Spearman Correlation Coefficient used as data were parametric.

The validation study identified minimal bias with POCT-Cr and eGFR, meaning no adaptations to the traffic light thresholds were required. For POCT-K, capillary samples had reduced accuracy, with a risk of overestimation, likely related to haemolysis. However, venous POCT-K underestimated laboratory results by 0.38 mmol/L. Therefore, we reduced the potassium concentration traffic light thresholds by 0.5 mmol/L to account for the systematic bias and included the differences in capillary and venous sampling results in the healthcare professional training.

A red-amber-green clinical decision support system (traffic light protocol) was used as this mechanism has previously been shown to provide clear guidance to healthcare professionals which translates to improved adherence to clinical guidelines, improved workflow efficiency and reduction in medication errors [25]. The clinical decision rules for the traffic light protocol are summarised in Table 2. Green outcome resulted in medication (RAASi or SGLT2i) increase or initiation. Amber outcome led to no immediate change. Red outcome resulted in dose reduction or cessation ± referral to the relevant services if severe. Three green domains were required for a green outcome. If one section was amber or red, then the outcome would be amber or red, respectively.

**Table 2.** Summary table for the traffic light clinical decision support system with associated rules and outcomes.

Parameter	Green outcome	Amber outcome	Red outcome
Systolic Blood Pressure (mmHg)	≥ 110	90–110	< 90 OR Symptomatic hypotension (e.g. dizziness when standing)
Percentage change in creatinine concentration	<30% change from baseline	30–-40% change from baseline	>40% change from baseline
Potassium concentration (mmol/L)^a^	Capillary: <5.5 Venous: <5.0	Capillary: 5.5–5.9 Venous: 5.0–5.4	Capillary: ≥6.0^b^ Venous: >5.5^c^

Green outcome = Medication initiation or dose increase. Amber outcome = no change in medication. Red outcome = Stop medication ± refer for blood test and clinical review.

^a^For potassium concentrations, red outcomes were divided into two levels. Level 1 = Stop Medication; Level 2 = Refer for venous blood test ± clinical review.

^b^Capillary potassium concentrations for level 1: 6.0–6.4 mmol/L; Level 2: ≥ 6.5 mmol/L.

^c^Venous potassium concentrations for level 1: 5.5–5.9 mmol/L; Level 2: ≥ 6.0 mmol/L.

### Clinic pathway

Clinics were conducted every 2–4 weeks during regular working hours, due to staff and room availability constraints. Further details of the clinic pathway are described in Supplementary Appendix A. Participants invited to the clinic received a care book developed for this clinic, containing their results and medication details. During their visit, they underwent a blood pressure check and POCT.

Based on the protocol outcome, medication decisions were communicated to the patient and documented in their care book, called ‘My Kidney Health’. This was provided to patients for personal use, containing only clinically relevant information (e.g. POCT results and medication changes) and remained the property of the patient. No data were extracted from the booklet, and no patient-identifiable information was included.

Follow-up appointments were scheduled every 2–4 weeks until pharmacological optimisation was achieved. Upon discharge, participants transitioned to routine follow-up care in accordance with local CKD guidelines.

### Healthcare personnel and training

Eligible patients were identified at each practice by a nephrologist using the APL CKD Tool, as previously described. The clinics were delivered by a nurse practitioner and/or healthcare assistant, with on-site support from a nephrologist during the initial phase, if issues were encountered.

Staff training was divided into three components: (1) Performing the POCT testing using the EPOC Blood Analysis System; (2) Interpreting results using the traffic light clinical decision support system and (3) Communicating the results and management plan to patients.

Each of these components was divided into one-hour sessions, with additional time allocated if required. Training continued during initial clinics, with an incremental increase in responsibility, aiming for independence after three clinics. Nurse practitioners were deemed competent pragmatically through direct observation during clinics. For example, performing the POCT test independently was determined by completing the tests during a clinic without hands-on support. Additional support was available upon request.

### Primary and secondary outcomes

Primary outcome was recruitment number and patient acceptability. Secondary outcomes were number (and proportion) of patients completing the pathway, the number of patients optimised, the number of visits/tests needed, prescribing and dose changes of RAASi; the number of failed tests, episodes of acute kidney injury and hyperkalaemia, the change in renal biochemistry analytes and the change in blood pressure.

### Data collection and extraction

Demographics, hypertension, diabetes mellitus and CKD diagnosis and medication doses were extracted from Primary Care records and entered by the project team into a password-protected project-specific database. All data were de-identified. Time from start to finish in pathway (days), start and end medications, number of medication changes and resource use (number of failed tests, time taken per visit) were also extracted from project logs.

All consultations were recorded using a standardised clinic template, which was only functional within the EMIS system's primary care electronic health record. The template was designed to provide simple and sustainable data extraction for this pilot and future audit purposes. However, no formal auditing or data monitoring was performed during the pilot pathway.

### Statistical analysis

This was a quantitative and supplementary qualitative analysis. Descriptive statistics were used to assess attendance, pathway completion, medication optimisation, recruitment rate (number of participants divided by the total number eligible to participate), number of visits, successful/failed POCT tests (reported as the proportion of successful tests against total attempts), and green/amber/red protocol outcomes. Did-not-attend (DNA) appointments were included as part of the visit analysis. Failed POCT test attempts were included in the denominator when calculating the success rate. However, no imputation was applied for missing biochemical data from unsuccessful tests.

This project was not powered to demonstrate a difference from standard care but would provide information on possible effect size to inform future evaluations. A CONSORT flow diagram was created to show number of participants approached, number attending each visit, the number of additional visits, the number of successful tests at six months and the reasons for non-optimisation, including loss-to-follow-up and withdrawal. All participants were invited to evaluate their experience of POCT-led monitoring using Likert scales and free-text responses. These responses were collated and analysed independently by two reviewers (RMG and KB). An inductive approach was used to assign initial codes to each response, which were then discussed and consolidated into themes. Given the small number and short nature of responses, the analysis was exploratory and aimed to identify recurring topics which supplement the quantitative findings.

Data were presented as counts and percentages with 95% confidence intervals were appropriate. Parametric data were presented as mean ± standard deviation, non-parametric data as median with interquartile range. Data were analysed using Software R V4.4.2.

All data were handled in accordance with information governance policies by responsible members of the local care team or project lead. Confidentiality was maintained by ensuring that any data used for service evaluation were anonymised prior to analysis.

## Results

### Pilot clinic pathway

Initial screening using the APL tool identified 89 patients in the three GP practices combined, who were shortlisted for detailed screening. Patients were excluded if they were already optimised (*n* = 35; 39.3%) or were deemed unsuitable for clinic (*n* = 6; 6.7%) for example if they were unable to travel, too frail or under palliative care. Out of the remaining 48 patients, eight (9.0%) declined to participate and 15 (16.9%) were not contactable, despite phone calls on three occasions.

The proportion of patients successfully recruited was 52% (25/48; 95% CI: 38.3% to 65.5%). Of those who declined to participate reasons included unable to attend (*n* = 1), apprehensive about changing medications (*n* = 2), needle phobic (*n* = 1) or not stated (*n* = 4). For patients who were not contactable, the mean age was 66.5 ± 12.3 years, 9 (60%) were from ethnic minority backgrounds and 10 (66.7%) were male.

**Figure 2.**
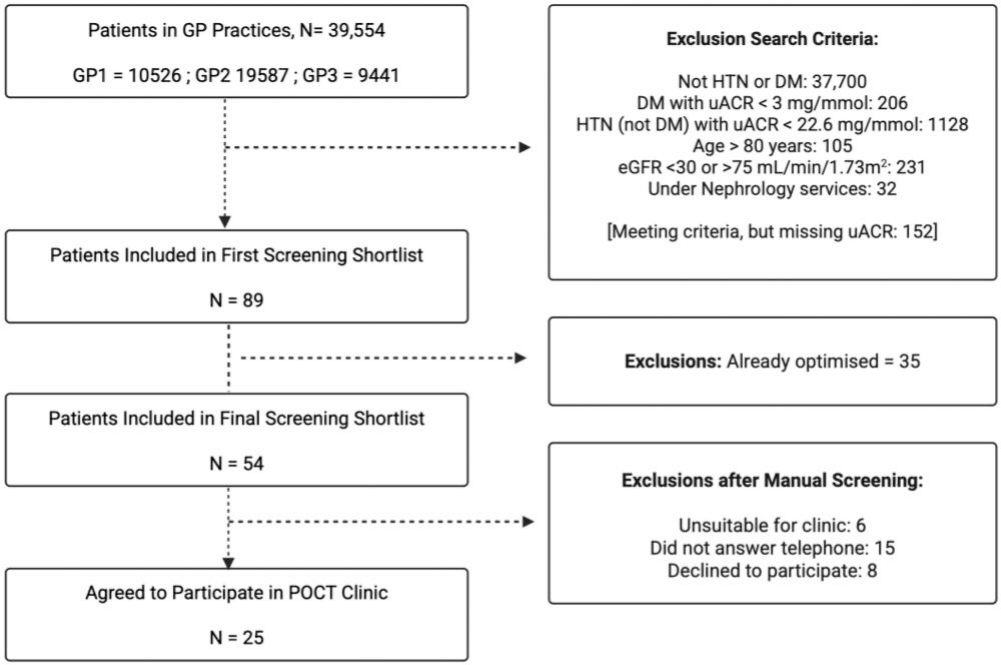
A CONSORT flow diagram illustrating the recruitment process for the pilot point-of-care testing Kidney Clinic in three Southeast London GP practices. Abbreviations: DM, Diabetes Mellitus; eGFR, Estimated glomerular filtration rate; GP, General Practice; HTN, Hypertension; POCT, point-of-care testing; uACR, Urinary albumin:creatinine ratio.

After exclusions 25 patients agreed to participate (see Fig. 2). Included participants were called up to two times prior to enrolment. There were 12 (48%) patients for SGLT2i initiation only, 2 (8%) for RAASi only and 11 (44%) for RAASi and SGLT2i initiation. Prior to starting the clinic 18 patients were already on RAASi (Candesartan = 5; Losartan = 3; Irbesartan = 2; Ramipril = 7; Lisinopril = 1).

Mean age was 64.5 ± 9.3 years and 17 were male (68%). Mean body mass index (BMI) was 32.0 ± 6.3 kg/m^2^. Most patients were from Black (*n* = 12; 48%) or White (n = 11; 44%) ethnicity backgrounds and just over half the patients had never smoked (*n* = 13; 52%).

At baseline, mean blood pressure was 134.6 ± 14.9/81.8 ± 10.8 mmHg, mean eGFR 56.7 ± 10.8 ml/min/1.73m^2^, mean creatinine concentration was 110 ± 25 micromol/L and potassium was 4.41 ± 0.51 mmol/L. Median urinary albumin: creatinine ratio was 13.7 (IQR 6.4–29.3). Baseline characteristics are summarised in Table 3.

**Table 3.** Baseline characteristics for patients included in the pathway.

Variable	Result
Number of participants	25
Mean Age ± SD, years	64.5 ± 9.3
Mean BMI ± SD, years	32.0 ± 6.3
Sex, *N* (%):	
Male	17 (68)
Female	8 (32)
Ethnicity, *N* (%):	
Black	12 (48)
White	11 (44)
South Asian	1 (4)
East Asian	1 (4)
Smoking History, *N* (%):	
Current smoker	7 (28)
Ex-smoker	5 (20)
Never smoked	13 (52)
Mean Baseline Blood Pressure ± SD, mmHg	134.6 ± 14.9/81.8 ± 10.8
Mean Baseline eGFR ± SD, mlL/min/1.73m^2^	56.7 ± 10.8
Mean Baseline Potassium ± SD, mmol/L	4.41 ± 0.51
Treatment Intent, *N* (%):	
RAASi only	2 (8)
SGLT2i only	12 (48)
RAASi and SGLT2i	11 (44)

Abbreviations: BMI, Body Mass Index; eGFR, Estimated glomerular filtration rate; RAASi, Renin angiotensin aldosterone system inhibitors; SGLT2i, sodium glucose co-transporter 2 inhibitors; SD, standard deviation.

**Figure 3.**
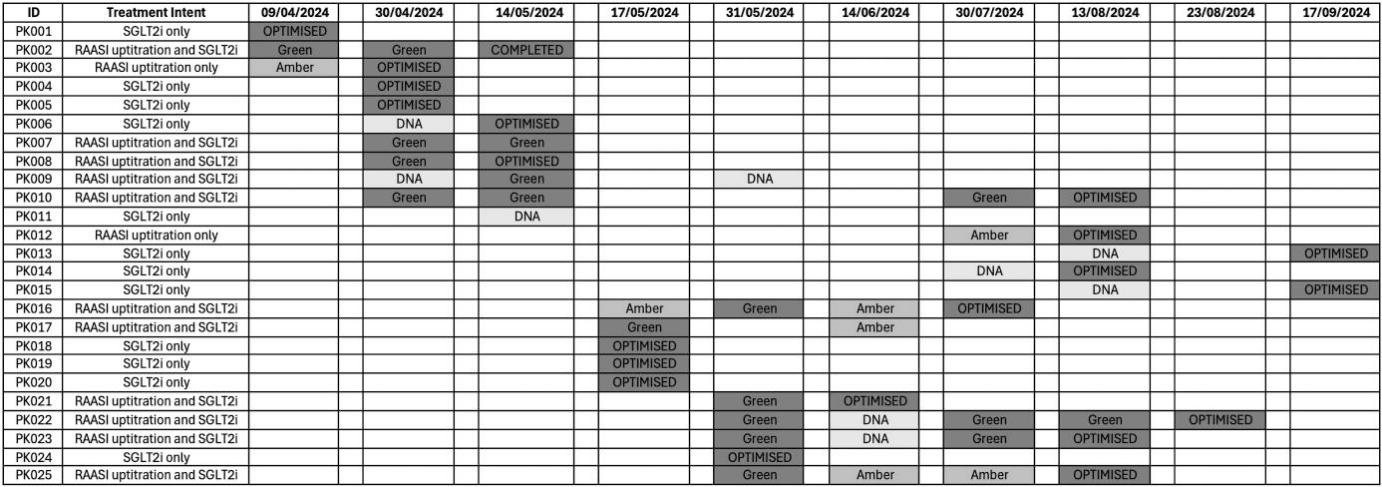
Gantt chart illustrating the timeline for clinic visits and traffic light protocol outcomes for each participant. Abbreviations: DNA, Did not attend; SGLT2i, sodium glucose cotransporter-2 inhibitor; RAASi, Renin−angiotensin−aldosterone system inhibitor. Each square represents a two-week time period. Treatment aim, did not attend and timing of optimisation is recorded. Clinic outcomes during each two-week period are highlighted in the corresponding colour of the clinical decision support system (green, amber or red).

There were 54 clinic appointments of which 8 (14.8%) were labelled as ‘Did Not Attend’ (DNA), due to patients not attending appointments. Mean duration between appointments was 25 ± 19 days. Overall, 23/25 (92%, 95% CI: 81–100%) patients completed the pathway and 20/25 (80%, 95% CI: 64–96%) were optimised. Mean duration from first clinic appointment to optimisation (excluding patients with single visits) was 46 ± 32 days. The pathway timeline for each participant is illustrated in Fig. 3.

**Figure 4.**
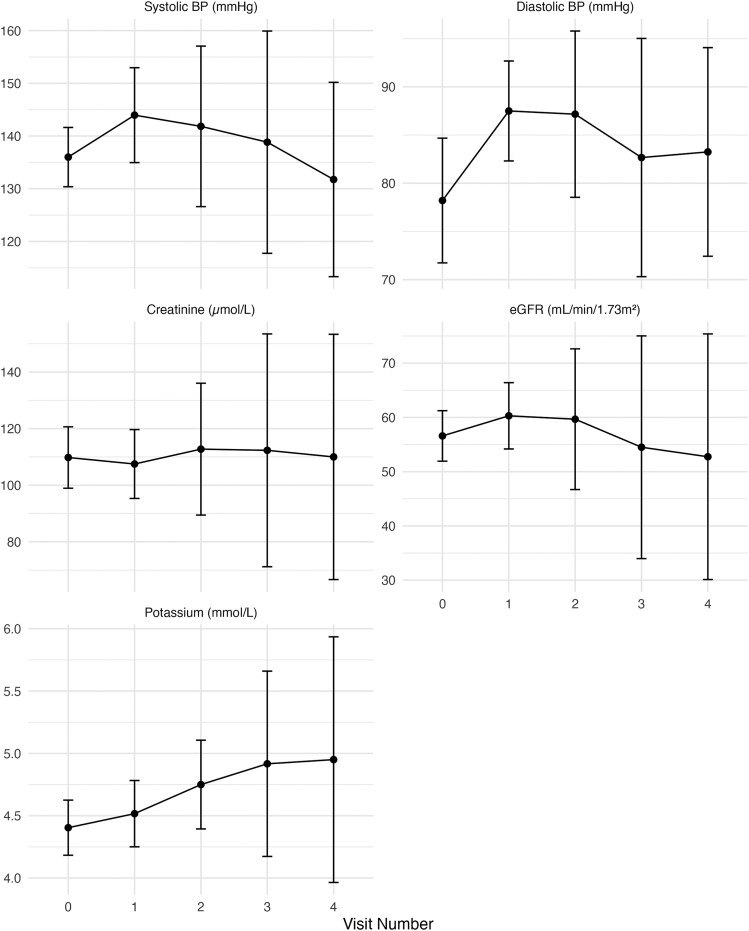
Series of line graphs illustrating the mean change with 95% confidence intervals in systolic blood pressure, diastolic blood pressure, creatinine concentration, eGFR and potassium concentration over each visit. Abbreviations: BP, Blood pressure; eGFR, Estimated glomerular filtration rate.

Thirty-nine (85%) protocol outcomes were green, 7 (15%) amber and none red, resulting in 39 medication changes (RAASi = 19; SGLT2i = 20). One patient had no medication changes despite a green outcome, as she declined the SGLT2i. Following RAASi up-titration, there were small changes in renal biochemistry between visits: Creatinine −2.7 ± 15.0 μmol/L (95% CI: −9.2 to 3.8 μmol/L), eGFR 2.82 ± 11.16 ml/min/1.73m^2^ (95% CI: −2.0 to 7.7 ml/min/1.73 m^2^) and potassium +0.04 ± 0.29 mmol/L (95% CI: −0.09 to 0.17 mmol/L). Changes in blood pressure and biochemistry between visits are illustrated in Fig. 4.

The majority of the samples taken were capillary (43/44; 97.7%). POCT tests were successful from 44/57 (77.2%, 95% CI: 66–88%) attempts. The main reason for unsuccessful attempts was nsufficient capillary sample size. There were no adverse events involving POCT. One patient developed haematuria and therefore stopped the SGLT2i whilst undergoing investigations.

An approximation of overall staff time required was 12 hours for screening, 9 hours for phone calls and administration and 39 hours for clinic visits. This equated to an average of 2.4 hours per participant.

There were 13 (52%) respondents to the questionnaire. Overall, 77% (10/13) were ‘very comfortable’ with finger-prick testing (Question 10), 69% (9/13) found receiving their results in clinic very useful (Question 11), 92% (12/13) questionnaire respondents were ‘very satisfied’ with their clinic experience (Question 14) and 69% (9/13) would recommend the clinic to others (Question 15). The majority indicated they would prefer an advanced nurse practitioner (*N* = 10) or pharmacist (*N* = 9) for future pathways.

Analysis of free-text comments from the patient questionnaires revealed multiple themes. First, several respondents reported that the clinic improved their understanding of their kidney health, with some noting this was the first time they received clear information about their kidney function. Secondly, participants generally accepted and valued the finger-prick testing approach, viewing it as a practical and desirable addition to general practice (‘The finger prick test struck me as something every GP should have available’). Finally, suggestions for improvement included establishing clearer follow-up communication and providing a dedicated point of contact for ongoing questions (‘There appears to be very little joined-up plan when somebody steps away’; ‘To have a contact to discuss any issues in the future’). This qualitative feedback reinforced the acceptability observed in the quantitative survey responses and provides valuable insights to inform future service improvements, particularly with addressing post-clinic lines of communication and support for patients.

## Discussion

Our protocolled POCT pathway demonstrated a high completion rate (92%) and pharmacological optimisation rate (80%), indicating that this model can be feasibly implemented in a primary care setting for people with early or advanced proteinuric CKD. This approach could strengthen current levels of care and improve cardiorenal protective medication prescribing rates. For example, OPTIMISE-CKD reported 31–59% of patients taking RAASi and only 2–3% taking SGLT2i in Japan, Sweden and the United States of America [26].

A major strength of this pathway is its ability to rapidly and efficiently up-titrate medications using POCT. Traditionally, the management of reno-protective agents in CKD is delayed due to serial blood test monitoring in overstretched services, resulting in delays or under-utilisation of RAASi and SGLT2i therapies. Integrating POCT into the pathway provided real-time decision making, expediting medication initiation and adjustment, and was more pragmatic in involving the patient in the management plan based on the results. These findings mirrored previous studies using POCT for monitoring renally excreted medications, with a Dutch study (*n* = 46), identifying over half requiring medication adjustments and positive experiences reported by patients [19].

POCT was well tolerated with a success rate of 77%. In addition, 77% of participants reporting feeling ‘very comfortable’ with finger-prick testing. This aligns with previous reports in the literature, with a UK-based mixed methods service evaluation reporting that patients receiving home visits with POCT benefitting from improved accessibility and reassurance with instant results [27]. In addition, there was high acceptability of the clinic, with 92% of participants being ‘very satisfied’ with their experience. These findings are consistent with a large Australian screening programme (KEY; n = 402), in which 99% of participants reported greater satisfaction and 96% reported improved understanding of their condition [16]. However, potential barriers to wider implementation include ensuring equitable engagement, workforce availability and initial device and consumable costs, which are discussed further in the limitations.

A framework combining a patient-centred (shared decision-making, supporting self-management and integrating care) and health systems approach (improving access to care and providing a decision support system) were utilised in this pathway through POCT and the traffic light protocol, which offered standardised and evidence-based decision-making support [28]. In this pilot, the traffic light protocol served as the decision support system. The majority of outcomes were classified as green (85%), indicating either medication up-titration or initiation of a new therapy, thereby facilitating rapid optimisation. There were no red outcomes, which is likely related to the inclusion criteria, which were based on national guidelines. The amber outcomes also highlight the benefits of early identification of changes in blood pressure or renal function, which would likely have an added benefit in monitoring of higher risk groups.

An additional objective of this pathway was to optimise pharmacological CKD therapies without adding further burden on primary care doctors. The pilot was run by an allied health professional; however, due to this being the first iteration, there was also a nephrology trainee doctor supporting the clinic visits. This approach was supported by most patients who preferred an advanced nurse practitioner or pharmacist to run future clinics.

Furthermore, in resource-limited and rural settings, POCT provides the added benefit of increased access for patients, who may otherwise have been facing longer journey times and/or delays in care. However, additional barriers include cost of stock distribution, POCT device maintenance, limited staffing and logistic limitations for on-site training [29, 30].

The integration of allied health professionals into CKD management complements wider healthcare policies towards multi-disciplinary management of long-term conditions and multi-morbidity. In the UK, this aligns with the NHS Long Term Plan which emphasises enhancing care in primary and community settings to reduce strain on hospital services and improve outcomes for people with chronic conditions. Empowering nurse practitioners, pharmacists, and other allied health professionals with greater responsibilities in co-leading CKD optimisation clinics, fosters collaboration within regional healthcare infrastructures and sets a foundation to expand into other specialties, such as heart failure or diabetes healthcare.

### Limitations

A limitation of this pilot was that the sample size was relatively small (*n* = 25). This is likely related to the identification component of the pathway only included patients who already have had renal function testing (renal profile and uACR) with their primary care team (coded or uncoded for CKD). A review of sample sizes for ISRCRN-registered UK pilot and feasibility studies reported a similar median target size of 30 (IQR 20–50) [31], however, to sustainably manage this pathway, increased recruitment would be required. Mechanisms to achieve this would be larger targeted testing (or screening programme) and increasing the patient population through operating at primary care network level.

In addition, 25.9% of patients were not contactable or declined participation. This is likely to have introduced selection bias thus overestimating outcomes, due to potential over-representation of individuals who are more likely to engage with primary care services. Many of the declining or unreachable patients were from ethnic minority groups (not contactable = 60%), indicating a barrier to engagement. This is likely indicative of underlying health inequalities, and therefore more detailed qualitative interrogation is needed to better understand ways to support those with challenges or hesitancy with engaging with healthcare services. In addition, equity-focussed strategies, such as targeted community engagement or culturally tailored communication may also be needed to improve inclusivity.

The pathway was implemented using existing staff capacity. Therefore, clinic scheduling was limited by staff availability, which may have influenced patient attendance, however, this was not formally assessed. This is a potential barrier to wider implementation of this pathway, as some practices may find additional staffing more challenging depending on existing workloads and local priorities. Additionally, a formal competency assessment was not undertaken, and in future scale-up, formal criteria will be needed. Finally, the initial cost of POCT devices and consumables could be prohibitive without start-up funding streams. A cost-saving analysis at this stage was not feasible as we were unable to evaluate practices not participating in the pilot, but long-term cost savings are anticipated due to reduced visits and expected improved cardiovascular and renal outcomes.

### Future direction

The next iteration of this pathway will include several additional objectives. Firstly, increasing the population size by running the clinics at larger scale (primary care network level). The increased recruitment through upscaling would also need to be balanced against increasing staff and resource requirements. Expansion to primary care network level would require increased governance to manage all participating practices, a range of clinic locations to cover a wider geographical area, increased consumables and staffing (which brings about greater cost) and the need for standardised training models with formal competence assessments. In resource-limited and rural settings, POCT provides the added benefit of increased access for patients, who may otherwise have been facing longer journey times and/or delays in care. However, additional barriers include cost of stock distribution, POCT device maintenance, limited staffing and logistic limitations for on-site training [29, 30].

Secondly, conducting semi-structured interviews and focus groups to provide a richer thematic analysis of patient and healthcare professional experience and perspectives on the pathway. Emphasis will be placed on acquiring greater understanding perspectives on CKD, POCT and preferences regarding monitoring and medication titration.

Thirdly, to assess whether there was any additional benefit for ethnic minority and socioeconomically deprived groups. Fourthly, to assess medium to long-term outcomes, such as cardiovascular events or need for dialysis, using long-term follow up data or modelling based on existing literature.

Finally, performing a business case report for the regional implementation at integrated care board level and a formal health economic evaluation to assess the cost-effectiveness and cost-utility of the POCT kidney clinic pathway using Markov models. Key metrics would include cost per patient optimised in the intervention compared to existing standard of care, modelled comparison of expected major cardiovascular events and initiation of dialysis stratified by CKD stage at time of intervention and projected quality of life years gained.

## Conclusion

This pilot demonstrates the feasibility and acceptability of a protocolled POCT pathway for optimising cardiorenal protective medications for people with proteinuric kidney disease and highlights a potential framework to improve access and efficiency in kidney disease management. High optimisation rates and patient satisfaction were reported but challenges remain regarding recruitment, engagement, and the assessment of sustainability and scalability.

## Supplementary Material

cmaf083_Supplementary_Data

## Data Availability

The individual-level raw data underlying this quality improvement project are not publicly available due to the inclusion of patient identifiable information and the terms of approval.
