# Supplementary material for: A pilot point-of-care kidney disease clinic in primary care to pharmacologically optimise people with chronic kidney disease (PROTECT KIDNEY)
Source: Fam Pract. 2025 Oct 30;42(6):cmaf083. doi: 10.1093/fampra/cmaf083 (PMC12573251; doi:10.1093/fampra/cmaf083)
Supplement: cmaf083_Supplementary_Data [file cmaf083_supplementary_data.pdf]

Supplemental Materials

Supplemental Appendix A: Methods - Clinic Pathway

Patients will have between 1-5 appointments until optimised. The clinic will run every 2-4 weeks, as per KDIGO recommendations for renal function testing following ACEi/ARB dose change. It will be run by an advanced nurse practitioner and/or healthcare assistant with initial support and training from a nephrology specialist trainee. Allied health professionals will be trained to use the device, interpret the results in line with the protocol and identify adverse events.

During a clinic visit, patients will be given information regarding CKD and its management, have a blood pressure and a finger-prick (capillary) or venous blood test for creatinine, eGFR and potassium as described above. Medication (ACEi/ARB/ SGLT2i) changes will be determined by the protocol.

An EMIS Health clinic template was created to standardise record keeping and simplify auditing. Documentation will include attendance versus Did Not Attend (DNA) and clinic visit number, treatment intent, blood pressure, POC-Cr, POC-K and eGFR, change in creatinine percentage, coded CKD stage, protocol outcome (Red, Amber or Green, medication change and dose ± whether optimised and the follow up or discharge plan.

Patients will be given a co-designed (with PPI input) care booklet called “My Kidney Health” to bring to each visit. This would record:

1. Baseline results (uACR, creatinine, eGFR, potassium, blood pressure)
2. Date of next clinic appointment
3. Creatinine result (umol/L)
4. Potassium result (mmol/L)
5. Blood pressure (mmHg)
6. Medication name and dose (including number of tablets)

Additional patient information will be provided with materials from Kidney Care UK (KCUK) and the National Health Service (NHS). Following pharmacological optimisation they would be discharged with routine follow up as per local and national guidelines.

Supplemental Figure 1. Illustrates four sections from the traffic light protocol: Capillary sample initial consultation algorithm; venous sample algorithm; medication dosing changes; adverse events regarding potassium and creatinine concentration results.

Capillary Sample: Initial Consultation

	Blood Pressure (BP)	Creatinine (Cr)	Potassium (K)	Outcome
CONSULTATION 1	Systolic BP ≥ 110 mmHg	< 30% increase from baseline Cr	< 5.5 mmol/L	Increase dose of ACEi / ARB<br>OR (if max)<br>Start SGLT2i
	Systolic BP 90 – 110 mmHg<br>AND<br>No dizziness on standing	30 – 40% increase from baseline Cr	5.5 – 5.9 mmol/L	Continue same dose of ACEi / ARB
	1/ Systolic BP < 90 mmHg<br>OR<br>Dizziness when standing	≥ 40% increase from baseline Cr result	1/ 6.0 – 6.4 mmol/L	1/ Stop Medication & Inform GP
			2/ ≥ 6.5 mmol/L	2/ Refer to A&E for blood test & Inform GP

Venous Sample: Initial Consultation

	Blood Pressure (BP)	Creatinine (Cr)	Potassium (K)	Outcome
CONSULTATION 1	Systolic BP ≥ 110 mmHg	< 30% increase from baseline Cr	< 5.0 mmol/L	Increase dose of ACEi / ARB<br>OR (if max)<br>Start SGLT2i
	Systolic BP 90 – 110 mmHg<br>AND<br>No dizziness on standing	30 – 40% increase from baseline Cr	5.0 – 5.4 mmol/L	Continue same dose of ACEi / ARB
	1/ Systolic BP < 90 mmHg<br>OR<br>Dizziness when standing	≥ 40% increase from baseline Cr result	1/ 5.5 – 5.9 mmol/L	1/ Stop Medication & Inform GP
			2/ ≥ 6.0 mmol/L	2/ Refer to A&E for blood test & Inform GP

Medication Dosing

Dose changes to be made in clinic are in bold

ACEi (Angiotensin-converting enzyme inhibitor)	Drug Name	Clinic Titration Doses → Max. tolerated dose (Once Daily)
	**Ramipril**	1.25mg → 2.5mg → **5mg** → 7.5mg → **10mg**
	Lisinopril	10mg → **20mg** → **40mg**
	Enalapril	5mg → 10mg → **20mg** → **40mg**
	Perindopril	2mg → **4mg** → 6mg → **8mg**
	Fosinopril	10mg → **20mg** → **40mg**
	Imidapril	5mg → **10mg** → **20mg**

OR

ARB (Angiotensin receptor blocker)	Drug Name	Clinic Titration Doses → Max. tolerated dose (Once Daily)
	**Irbesartan**	75mg → **150mg** → **300mg**
	Losartan	25mg → **50mg** → **100mg**
	Valsartan	80mg → **160mg** → 240mg → **320 mg**
	Candesartan	4mg → 8mg → 12mg → **16mg** → 24mg → **32mg**
	Olmesartan	10mg → **20mg** → **40mg**
	Telmisartan	20mg → **40mg** → **80mg**

AND

SGLT2i (Sodium Glucose Co-transporter 2 inhibitor)	Drug Name	Clinic Titration Doses → Max. tolerated dose (Once Daily)
	Dapagliflozin	10mg
	Empagliflozin	10mg

Adverse Events: Potassium + Creatinine

1. High Potassium

Hyperkalaemia (high potassium) is a medical emergency, which can be life-threatening.

Using the EPOC POC device, if Potassium:
≥ 6.0 mmol/L (Capillary)
≥ 5.5 mmol/L (Venous)
Plan: **STOP the ACEi or ARB** (see medications) and to **attend A&E (if out of hours) for a venous blood test**.

2. Low Potassium

Hypokalaemia (low potassium) is also a medical emergency as it can affect the electrical conduction of the heart.

Using the EPOC POC device, if Potassium:
< 3.5 mmol/L (Capillary)
< 3.1 mmol/L (Venous)
Plan: **Up-titrate ACEi or ARB if x3 green** and **Refer to GP** and **organise venous blood test**

3. Declining Renal Function / Acute Kidney Injury

If a patient has 2 sequential rises in creatinine (AMBER or RED) they should stop their ACEi / ARB and be booked in to see the GP.

See next page for AKI guideline.

Standards for QUality Improvement Reporting Excellence (SQUIRE 2.0) Checklist

Text section and item name	Page/line no(s).
	info is located
**Title and abstract**	
1. **Title**	
Indicate that the manuscript concerns an initiative to improve healthcare (broadly defined to include the quality, safety, effectiveness, patient-centredness, timeliness, cost, efficiency and equity of healthcare).	Page 1
2. **Abstract**	
a. Provide adequate information to aid in searching and indexing.	Page 2
b. Summarise all key information from various sections of the text using the abstract format of the intended publication or a structured summary such as: background, local problem, methods, interventions, results, conclusions.	Pages 2 - 3
**Introduction: Why did you start?**	
3. **Problem description** - Nature and significance of the local problem.	Pages 4-5
4. **Available knowledge** - Summary of what is currently known about the problem, including relevant previous studies.	Page 4-5
5. **Rationale** - Informal or formal frameworks, models, concepts and/or theories used to explain the problem, any reasons or assumptions that were used to develop the intervention(s) and reasons why the intervention(s) was expected to work	Page 5
6. **Specific aims** - Purpose of the project and of this report.	Page 5
**Methods: What did you do?**	
7. **Context** - Contextual elements considered important at the outset of introducing the intervention(s).	Page 5
8. **Intervention(s)**	
a. Description of the intervention(s) in sufficient detail that others could reproduce it.	Pages 5-10
b. Specifics of the team involved in the work.	Pages 9-10
9. **Study of the intervention(s)**	
a. Approach chosen for assessing the impact of the intervention(s).	Pages 5, 10-11
b. Approach used to establish whether the observed outcomes were due to the intervention(s).	Page 11
10. **Measures**	
a. Measures chosen for studying processes and outcomes of the intervention(s), including rationale for choosing them, their operational definitions and their validity and reliability.	Page 10

b. Description of the approach to the ongoing assessment of contextual elements that contributed to the success, failure, efficiency and cost.	Page 6
c. Methods employed for assessing completeness and accuracy of data.	Pages 11-12
11. **Analysis**	Pages 11-12
a. Qualitative and quantitative methods used to draw inferences from the data.	Pages 11
b. Methods for understanding variation within the data, including the effects of time as a variable.	Pages 11-12
12. **Ethical considerations** - Ethical aspects of implementing and studying the intervention(s) and how they were addressed, including, but not limited to, formal ethics review and potential conflict(s) of interest.	Page 19
**Results: What did you find?**	
13. **Results**	
a. Initial steps of the intervention(s) and their evolution over time (eg, time-line diagram, flow chart or table), including modifications made to the intervention during the project.	Page 25 (Figure 3)
b. Details of the process measures and outcomes.	Pages 12 - 15
c. Contextual elements that interacted with the intervention(s).	Pages 12 - 15
d. Observed associations between outcomes, interventions and relevant contextual elements.	Pages 14 - 15
e. Unintended consequences such as unexpected benefits, problems, failures or costs associated with the intervention(s).	Pages 17-18
f. Details about missing data.	Page 11
**Discussion: What does it mean?**	
14. **Summary**	
a. Key findings, including relevance to the rationale and specific aims.	Pages 14 - 16
b. Particular strengths of the project.	Pages 14 - 15
15. **Interpretation**	
a. Nature of the association between the intervention(s) and the outcomes.	Pages 15 - 17
b. Comparison of results with findings from other publications.	Pages 15 - 17
c. Impact of the project on people and systems.	N/A
d. Reasons for any differences between observed and anticipated outcomes, including the influence of context.	Page 17
e. Costs and strategic trade-offs, including opportunity costs.	Pages 14 & 18
16. **Limitations**	
a. Limits to the generalisability of the work.	Pages 17 - 18
b. Factors that might have limited internal validity such as confounding, bias or imprecision in the design, methods, measurement or analysis.	Pages 17 - 18
c. Efforts made to minimise and adjust for limitations.	Page 17 - 18
**Conclusions**	

a. Usefulness of the work.	Pages 15-17, 19
b. Sustainability.	Pages 18 - 19
c. Potential for spread to other contexts.	Page 17
d. Implications for practice and for further study in the field.	Pages 17
e. Suggested next steps.	Pages 18 - 19
**Other information**	
18. **Funding** - Sources of funding that supported this work. Role, if any, of the funding organisation in the design, implementation, interpretation and reporting.	Page 20
